# Insights into the evolution of sialic acid catabolism among bacteria

**DOI:** 10.1186/1471-2148-9-118

**Published:** 2009-05-26

**Authors:** Salvador Almagro-Moreno, E Fidelma Boyd

**Affiliations:** 1 Department of Biological Sciences, University of Delaware, Newark, DE 19716, USA; 2 Department of Microbiology, National University of Ireland, University College Cork, Cork, Ireland

## Abstract

**Background:**

Sialic acids comprise a family of nine-carbon amino sugars that are prevalent in mucus rich environments. Sialic acids from the human host are used by a number of pathogens as an energy source. Here we explore the evolution of the genes involved in the catabolism of sialic acid.

**Results:**

The cluster of genes encoding the enzymes *N*-acetylneuraminate lyase (NanA), epimerase (NanE), and kinase (NanK), necessary for the catabolism of sialic acid (the Nan cluster), are confined 46 bacterial species, 42 of which colonize mammals, 33 as pathogens and 9 as gut commensals. We found a putative sialic acid transporter associated with the Nan cluster in most species. We reconstructed the phylogenetic history of the NanA, NanE, and NanK proteins from the 46 species and compared them to the species tree based on 16S rRNA. Within the NanA phylogeny, Gram-negative and Gram-positive bacteria do not form distinct clades. NanA from *Yersinia *and *Vibrio *species was most closely related to the NanA clade from eukaryotes. To examine this further, we reconstructed the phylogeny of all NanA homologues in the databases. In this analysis of 83 NanA sequences, Bacteroidetes, a human commensal group formed a distinct clade with Verrucomicrobia, and branched with the Eukaryotes and the *Yersinia/Vibrio *clades. We speculate that pathogens such as *V. cholerae *may have acquired NanA from a commensal aiding their colonization of the human gut. Both the NanE and NanK phylogenies more closely represented the species tree but numerous incidences of incongruence are noted. We confirmed the predicted function of the sialic acid catabolism cluster in members the major intestinal pathogens *Salmonella enterica, Vibrio cholerae, V. vulnificus, Yersinia enterocolitica *and *Y. pestis*.

**Conclusion:**

The Nan cluster among bacteria is confined to human pathogens and commensals conferring them the ability to utilize a ubiquitous carbon source in mucus rich surfaces of the human body. The Nan region shows a mosaic evolution with NanA from *Bacteroidetes, Vibrio *and *Yersinia *branching closely together with NanA from eukaryotes.

## Background

**Figure 1:**
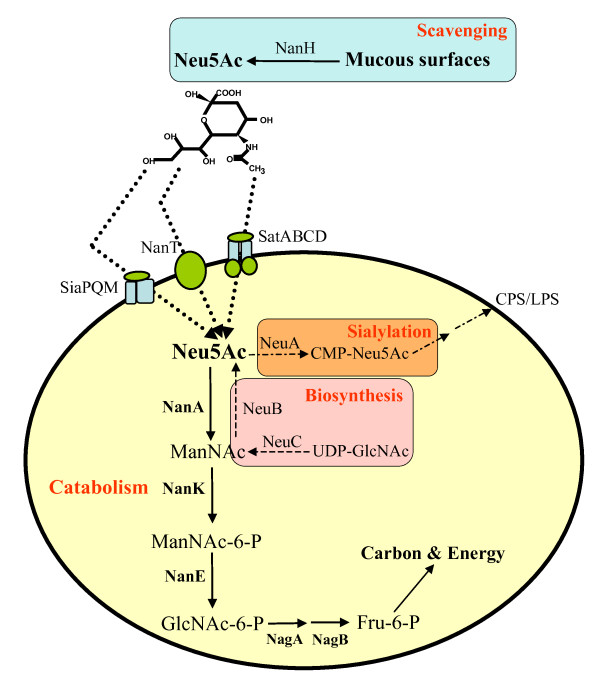
**Schematic representation of the metabolism of sialic acid among Bacteria**. Summary of varied pathways of sialic acid utilization in Bacteria. The catabolic pathway of sialic acid involves several steps beginning with NanA. Highlighted in orange is the donor-scavenging synthesis of sialic acid. Highlighted in pink is the de novo pathway for the synthesis of sialic acid. For a more comprehensive review of sialic acid utilization see refs. 1 and 29. NanH, Neuraminidase; Neu5Ac, *N*-acetylneuraminic acid, sialic acid; T, sialic acid transporter; NanA, *N*-acetylneuraminic acid lyase; ManNAc, *N*-acetylmannosamine; NanK, *N*-acetylmannosamine kinase; ManNAc-6-P, *N*-acetylmannosamine-6-phosphate; NanE, *N*-acetylmannosamine-6-P epimerase; GlcNAc-6-P, *N*-acetylglucosamine-6-phosphate; NagA, *N*-acetylglucosamine-6-phosphate deacteylase; GlcN-6-P, Glucosamine-6-phosphate; NagB, Glucosamine-6-phosphate deaminase; Fru-6-P, Fructose-6-phosphate; NeuA, CMP-*N*-acetylneuraminic acid synthetase; CMP-NeuAc, CMP-*N*-acetylneuraminic acid; NeuB, *N*-acetylneuraminic acid synthase; LPS, lipopolysaccharide.

Sialic acid or neuraminic acid, is the designation of a family that encompasses over 50 naturally occurring and structurally distinct nine-carbon amino sugars found both in the Eukaryotes and Prokaryotes, being the only nine-carbon sugar known to date in the latter [1]. Both names, sialic acid and neuraminic acid, indicate the source of the molecules from which they were first discovered: sialic, saliva in Greek, and neuraminic, brain and amine [2]. The most abundant and widely studied sialic acid is *N*-acetylneuraminic acid (2-keto-3-deoxy-5-acetamido-D-glycero-D-galacto-nonulosonic acid or Neu5Ac), with the rest of the sialic acids being derivatives of Neu5Ac (Fig. 1) [1-4].

In eukaryotes, sialic acids are primarily found at terminal positions of numerous glycoconjugates, and are involved in a varied array of cell-cell interactions and cell-molecule recognition, such as stabilizing glycoconjugates and cell membranes, or acting as chemical messengers [5,6]. Thus, the presence of sialic acid is crucial for the development of vertebrates, with mutations in the synthesis pathway causing premature death of mice embryos [7]. Sialic acids are widely found in Deuterostomes and recent speculation suggests that they might appear in particular life stages or in small quantities in Protostomes [8-10]. Sialic acids are also found in Fungi and some protozoa, although the latter likely can only scavenge them from the host [11-13].

Current studies have shown that several bacterial pathogens such as enterohemorrhagic *Escherichia coli*, *Haemophilus influenzae*, *H. ducreyi*, *Pasteurella multocida*, *Neisseria gonorrhoeae*, *N. meningitidis*, *Campylobacter jejuni*, and *Streptococcus agalactiae *can put sialic acid residues on their outer surfaces (sialylate) masking them from the host immune system [14-32]. Interestingly, these pathogens have developed different mechanisms for obtaining sialic acid that include de novo biosynthesis of sialic acid (*E. coli, N. meningitidis*), sialic acid scavenging (*N. gonorrhoeae*), or precursor scavenging (*H. influenzae*) (Fig. 1) [14-17].

Bacteria can also utilize sialic acid as a carbon and nitrogen source by scavenging it from the surrounding environment [1,18-23]. The catabolic pathway of sialic acid in bacteria involves five steps (Fig. 1): first *N*-acetylneuraminic lyase (NanA) removes a pyruvate group from Neu5Ac yielding *N*-acetylmannosamine (ManNAc), and then *N*-acetylmannosamine kinase (NanK) adds a phosphate group at C6 position, which yields *N*-acetylmannosamine-6-P (ManNAc-6P). Next, *N*-acetylmannosamine-6-P epimerase (NanE) epimerizes the ManNAc-6P into *N*-acetylglucosamine-6-P (GlcNAc-6P). Then, *N*-acetylglucosamine-6-P deacetylase (NagA) removes the acetyl group from GlcNAc-6P and yields Glucosamine-6-P (GlcN-6P) whose amine group is removed by Glucosamine-6-P deaminase (NagB), which is converted into Fructose-6-P (Fig. 1) [19,24-28]. Interestingly, recent studies have shown an alternative in the catabolic pathway of the gut commensal *Bacteroides fragilis*, which does not encode NanK since the epimerase (NanE) does not require a phosphorilated substrate to perform its metabolic reaction [23]. The genes encoding NanA, NanK, and NanE are clustered together, however the location of the genes that encode NagA and NagB is highly variable, in some cases being part of the cluster, such as in *H. influenzae*, or in most cases, scattered in the genome [1]. For this reason, in this work we focused on *nanA*, *nanK*, and *nanE*, which from now on we will refer to as the Nan cluster.

Few studies have investigated the ability to utilize sialic acid as a carbon source or its relationship to bacterial pathogenesis, even though the molecule is extensively found in mucus rich environments such as the gut and lungs where many pathogens thrive [18,21-23,28,29]. Sialic acid catabolism has been demonstrated in five species: *Clostridium perfringens*, *E. coli*, *P. multocida*, *H. influenzae*, and *Bacteroides fragilis *[21-23,28,29]. Chang *et al *showed in *E. coli *that the ability to degrade sialic acid was important for the colonization of the mouse colon [18]. This finding suggests that the ability to utilize sialic acid as a carbon source may be important for bacteria to colonize this niche. To date, little is known about the distribution of the Nan genes in the Bacterial kingdom and their evolutionary history [1,30,31]. de Koning and colleagues in their analysis of inter domain transfer events found that lateral gene transfer of *nanA *had occurred between bacteria and the human parasite *Trichomonas vaginalis *based on the phylogeny of 15 *N*-acetylneuraminate lyase protein sequences [31]. Furthermore, their analysis also indicated possible transfer between Gram-negative and Gram-positive bacteria.

In this study, we examined the distribution of the Nan cluster (*nanA, nanE *and *nanK*) among the 1,902 bacterial genomes in the database and found that these genes have an extremely limited distribution. The Nan cluster is confined to predominantly pathogenic and commensal bacteria. The cluster was present only in members of the Gamma-Proteobacteria and Fusobacteria among Gram-negative bacteria, and Bacillales, Clostridia, and Lactobacillales among Gram-positive bacteria as well as Mycoplasma. We studied the gene order of the cluster, and uncovered a surprising variability in its organization even within members of the same genus. We identified a putative sialic acid transporter within the Nan clusters from 40 of the 46 species that contained the region. We describe four novel sialic acid transporter types and found different transporters associated with Nan within each major phylogenetic group. We reconstructed the phylogenetic relationships of NanA, NanE, and NanK and demonstrated that NanA evolved independently from NanE and NanK. The NanA phylogenetic tree in particular revealed several putative horizontal gene transfer events, one involving transfer between domains. To examine further the evolution of *N*-acetylneuraminate lyase (NanA), the key enzyme in sialic acid catabolism, we determined the distribution and phylogeny of all homologues in the database. NanA was present in four additional bacterial groups, α-Proteobacteria, Planctomycetes, Verrucomicrobia and Bacteroidetes. The Bacteroidetes, members of the human gut microbiota, formed a distinct clade with the Verrucomicrobia and this clade is most closely related to the eukaryotes. The *Vibrio/Yersinia *clade branched next to the Bacteroidetes clade. We verified that sialic acid can be used as a sole carbon source in a number of pathogenic species, a capability that should confer a competitive advantage in the heavily sialylated environments of the human body.

## Methods

### Sequence retrieval and cluster identification

BLAST searches were used to identify homologues of NanA, NanE, and NanK from *Vibrio cholerae *N16961 and *Staphylococcus aureus *N315 in the database. We considered three genes to form a Nan cluster if: 1) they were the best matches in BLAST for NanA, NanE, and NanK from *V. cholerae *N16961; 2) reciprocal BLAST searches of the three genes against the *V. cholerae *N16961 genome hit on *V. cholerae *N16961 NanA, NanE, and NanK; 3) the same applied when performing BLAST search using NanA, NanK, and NanE from *S. aureus *N315; 4) the three genes were encoded and within 10 ORFs of each other. The rationale for this approach was: A) to avoid false positives, such as dihydropicolinate synthase, which shows similarity with NanA, and several sugar kinases that were retrieved when we searched for NanK homologues; and B) BLAST search using *S. aureus *N315 allowed us to avoid false negatives due to the low similarity of *S. aureus *Nan genes with the Nan genes from *V. cholerae *N16961.

The DNA sequences of the 16S rRNA from species encoding the Nan cluster were downloaded from GenBank. When several strains from the same species encoded the same cluster only a representative strain was included, with the strain from the first sequenced genome chosen.

### Sialic acid transporter identification

In order to identify putative sialic acid transporters within the Nan cluster we used the NanT protein from *E. coli *K12 (b3224), the DctM protein component from the TRAP transporter in *V. cholerae *(VC1777), and the SatB protein from the SatABCD transporter from *H. ducreyi *(HD1670) as seeds in our BLAST searches [32-34]. In addition, we also examined the distribution of sialic acid biosynthesis genes (*neuA, neuB*, and *neuC*) among the bacteria encoding the Nan cluster to determine whether there was a correlation between the presence of genes involved in sialic acid catabolism and the genes required for the synthesis of sialic acid de novo.

### Sequence alignment

The sequences were aligned using ClustalW [35]. The alignments were further checked manually using GeneDoc [36]. Large gaps and hypervariable sites were removed from the alignments; the same was applied to gaps at the beginning and end of the alignment, representing missing sequence data.

### Phylogenetic analysis

We used prottest and modeltest (protein and DNA sequences respectively) in order to choose the most appropriate method to calculate the distances [37]. We chose WAG with invariable sites for the Nan protein sequences and GTR with invariable sites for 16S rRNA sequences [38,39]. Three different tree-building methods were used: Maximum Likelihood (ML), Bayesian analysis (BY), and Neighbor Joining (NJ) as implemented in PHYML and MrBayes 3.1.2, and MEGA 4 respectively [40-43]. The Bootstrap values for ML and NJ trees were obtained after 1000 generations. For the trees constructed using BY the Markov chains were run for 1,000,000 generations. The burn-in values were set for 10,000 generations and the trees were sampled every 100 generations. Splitstree and MEGA 4 tree viewer were used to visualize the trees and calculate confidence values [43,44]. The topology of ML, NJ and BY trees were very similar, with differences in branch lengths and confidence values but not in branching pattern. NJ and ML trees are included as additional files (see Additional files 1, 2, 3, 4, 5 and 6).

### GC content

We calculated the GC content of the sequences and compared it to the GC content of the whole genome. The formulae used for the calculations can be found in Karlin *et al*., 2001 [45] (see Additional file 7).

### Growth analysis of various species on minimal media supplemented with sialic acid

We inoculated 2% (100 μl) of a 5 ml overnight LB broth culture into 5 ml of M9 minimal media (*V. cholerae *N16961, *V. vulnificus *YJO16, *V. parahaemolyticus *RIMD, *V. fischeri *H905, *Salmonella enterica *INSP85, MOPS-based minimal media (*Yersinia pestis *KIM D27), or M9 minimal media supplemented with amino acids (*Yersinia enterocolitica *ATCC 27729) [46]. All media were supplemented with 1 mg/ml of *N*-acetylneuraminic acid (Sigma) or D-Glucose. The growth of each species was detected by measuring the absorbance of the cultures at 595 nm using a Sunrise 96-well plate reader by Tecan. The incubation temperature was 30°C for all the species except for *Y. pestis *which was incubated at 26°C. The data obtained was exported to an excel sheet, and the growth curves were made using Sigma plot.

## Results and Discussion

### Distribution of Nan cluster

The distribution of the Nan cluster was remarkable in its limited occurrence among the 730 finished and 1172 unfinished bacterial genomes examined. The Nan cluster was present in 46 bacterial species and confined to six bacterial families of the Gamma-Proteobacteria, one member of the genus *Fusobacterium*, and five bacterial families of the Firmicutes, encompassing both low and high GC representatives (see Additional file 8).

Interestingly, apart from *Photobacterium profundum*, *Pseudoalteromonas haloplanktis*, *Shewanella pealeana*, *Psychromonas*, and *Vibrio*, which are all aquatic bacteria, the Nan cluster is only present in either commensal or pathogenic bacteria (see Additional file 8). In fact, 42 species from the 46 where the Nan cluster was identified are human commensals or pathogens, 33 are known pathogens of either humans or livestock. A total of 43% of the genomes in the databases belong to pathogens, whereas 72% of the species that contain the Nan clusters are pathogenic. The pathogens that encode the cluster are causative agents of a wide range of diseases; many of them are intestinal pathogens such as *E. coli, Shigella, Salmonella enterica, Yersinia enterocolitica, V. vulnificus *and *V. cholerae*, the etiological agent of cholera. *Clostridium botulinum *is the causative agent of botulism, *Haemophilus influenzae *is a major cause of lower respiratory infections and meningitis in children, *Streptococcus pneumoniae *causes pneumonia, and *Yersinia pestis *is the agent of plague. The nine human gut commensals that encode the Nan cluster include *Anaerotruncus colihominis*, *Dorea formicigenerans*, *D. longicatena, Faecalibacterium prausnitzii*, *Fusobacterium nucleatum, Ruminococcus gnavus*, *Lactobacillus sakei, L. plantarum*, and *L. salivarius*. Thus, the majority of the bacteria that encode the Nan cluster colonize mucous regions of the human body, such as the gut, lung, bladder, or oral cavity, where sialic acid is highly abundant and it can serve as a source of energy, carbon, and nitrogen.

We also examined the intraspecies distribution of the Nan cluster to determine whether all strains from a species encoded the cluster. We found that for most species all sequenced strains contained the Nan cluster. However, a few exceptions were noted. For example, of the eight fully sequenced genomes of *C. botulinum*, only strain Eklund encoded the Nan cluster. Among the six sequenced *S. pneumoniae *strains, five (TIGR, D39, G54, Hungary 19 A-6, and R6) encoded two Nan clusters whereas strain CGSP14 does not contain the cluster. Similarly, for *Salmonella enterica*, two out of the nine sequenced strains do not encode the Nan cluster (serovar Typhi Ty2 and CT18). Among the 16 sequenced *V. cholerae *isolates, only the 10 sequenced pathogenic isolates encoded the Nan cluster.

To make the study tractable, we took the approach to include in our analysis only the species that encoded the three genes *nanA*, *nanK*, and *nanE *(the Nan cluster) which were within 10 ORFs from each other. In addition, we investigated the number of species that encoded NanA, the key enzyme in the first step of sialic acid degradation, but did not encode NanK or NanE. Overall, the distribution of NanA, from this analysis resembles that of the Nan cluster with only four additional bacterial groups added, two genera from α-Proteobacteria, and several members of the Planctomycetes, Verrucomicrobia and Bacteroidetes. Interestingly, members of the *Bacteroides *are well known commensals of the human gut that in some cases can become opportunistic pathogens. The mucinolytic abilities of the *Bacteroides *have been documented, and sialic acid seems to be an important carbon source for these organisms [23,47]. However, *Bacteroides *does not require the presence of NanK in order to catabolize sialic acid [23]. The Verrucomicrobia are a recently described phylum of Bacteria and are recovered from fresh water, soil and human feces. Overall, the majority of the additional species identified as containing NanA were commensal or pathogens of humans.

### Sialic acid transporters within the Nan cluster

Prior to its catabolism, sialic acid has to be transported into the cell, unless there is endogenous biosynthesis (Fig. 1). To date there are three functionally characterized sialic acid transporters: NanT, a single component system, which belongs to the major facilitator superfamily, first identified in *E. coli*; a tripartite ATP-independent periplasmic C4-dicarboxilate (TRAP) multicomponent transport system, first identified in *H. influenzae *and *Pasteurella multocida*; and an ATP binding cassette (ABC) transporter, first identified in *Haemophilus ducreyi *[1,3,48]. We identified a putative sialic acid transporter in 40 of the 46 species examined, four of the seven families of transporters were novel types associated with sialic acid (Table 1).

**Table 1:** Sialic acid transporters within Nan cluster

Species	type	locus	e-value
*Clostridium botulinum *C str. Eklund	SSS	CBC_A1223	4.00E-78
*Clostridium perfringens *SM101	SSS	CPR_0176	6.00E-63
*Dorea formicigenerans *ATCC 27755	SSS	DORFOR_00337	4.00E-60
*Dorea longicatena *DSM 13814	SSS	DORLON_02532	2.00E-59
*Escherichia coli *K12	NanT	b3224	seed
*Escherichia coli *O157:H7 str. Sakai	NanT	ECs4097	0
*Fusobacterium nucleatum *ATCC 25586	TRAP	FN1473	2.00E-105
*Haemophilus influenzae *Rd KW20	TRAP	HI0147	8.00E-103
*Haemophilus somnus *129PT	TRAP	HS_0702	3.00E-104
*Lactobacillus plantarum *WCFS1	SSS	lp_3563	8.00E-96
*Lactobacillus sakei subsp*. 23K	SSS	LSA1642	4.00E-96
*Mycoplasma capricolum *ATCC 27343	SSS	MCAP_0416	1.00E-75
*Mycoplasma mycoides *SC str. PG1	SSS	MSC_0558	1.00E-74
*Mycoplasma synoviae *53	SSS	MS_0191	1.00E-45
*Pasteurella multocida *subsp.*Multocida *Pm70	TRAP	PM1708	2.00E-101
*Photobacterium profundum *SS9	TRAP	PBPRA2279	5.00E-169
*Pseudoalteromonas haloplanktis *TAC125	SSS	PSHAb0151/0160	1e-75/6e-75
*Salmonella enterica *ser.*Cholerasuis *SC-B67	NanT	SC3276	0
*Salmonella enterica *ser.*Paratyphi *A str. ATCC 9150	NanT	SPA3206	0
*Salmonella typhimurium *LT2	NanT	STM3338	0
*Shewanella pealeana *ATCC 700345	SSS	Spea_1518	3.00E-78
*Shigella boydii *Sb227	NanT	SBO_3165	0
*Shigella dysenteriae *Sd197	NanT	SDY_3399	0
*Shigella flexneri *2a str. 301	NanT	SF3260	0
*Shigella sonnei *Ss046	NanT	SSO_3365	0
*Staphylococcus aureus subsp. Aureus *N315	Sym	SA0303	0
*Staphylococcus haemolyticus *JCSC1435	Sym	SH0283	0
*Staphylococcus saprophyticus *ATCC 15305	Sym	SSP0376	seed
*Streptococcus agalactiae *2603V/R	SAT 3	SAG0035	4.00E-121
*Streptococcus gordonii str*. Challis substr. CH1	SAT 2	SGO_0122	1.00E-130
*Streptococcus pneumoniae *TIGR4 a	SAT 2/3	SP_1688 SP_1682	3.00E-131
*Streptococcus pneumoniae *TIGR4 b	Sym	SP_1328	8.00E-59
*Streptococcus pyogenes *M1 GAS	SAT 3	Spy_254	seed
*Streptococcus sanguinis *SK36	SAT 2	SSA_0076	seed
*Vibrio cholerae *N16961	TRAP	VC1777	seed
*Vibrio fischeri *ES114	SSS	VF0668	seed
*Vibrio vulnificus *YJ016	TRAP	VVA1200	0
*Yersinia bercovieri *ATCC 43970	NanT	YberA_01002486	1.00E-91
*Yersinia enterocolitica subsp. enterocolitica *8081	NanT	YE1193	0
*Yersinia frederiksenii *ATCC 33641	NanT	YfreA_01001471	0
*Yersinia mollaretii *ATCC 43969	NanT	YmolA_01003560	0
*Yersinia pestis *KIM	NanT	y1465	1.00E-180
*Yersinia pseudotuberculosis *IP 32953	NanT	YPTB2736	0

NanT (b3224) Major facilitator superfamily sialic acid transporter

TRAP (VC1777) Tripartite ATP-independent periplasmic C4-dicarboxylate transport system

SAT (HD1670) ATP binding cassette transporter

SAT 2 (SSA_0076) ATP binding cassette transporter

SAT 3 (Spy_254) ATP binding cassette transporter N-acetylneuraminate transport system permease

SSS (VF0668) Sodium-glucose/galactose cotransporter/SSS family

Sym (SSP0376) Na+/proline symporter

All the Gamma-Proteobacteria that contained the Nan cluster encoded one of three types of transporters within the cluster: homologues of NanT, TRAP, or a novel Sodium-glucose/galactose cotransporter, which belongs to the SSS family of transporters (Table 1). The three members of the Pasteurellaceae encoded the multicomponent TRAP transporter system, whereas all the Enterobacteriaceae examined encoded the NanT single component transporter, similar to that present in *E. coli *(Table 1). All members of the family Vibrionaceae, except *V. fischeri*, encoded a TRAP transporter. Within the Nan cluster among the Firmicutes, the predominant transporter associated with the Nan cluster belonged to SSS, ABC, or Sodium/proline (Sym) family of transporters. None of the Firmicute representatives examined encoded either the NanT or the TRAP systems.

### Co-occurrence of catabolic and biosynthetic sialic acid gene clusters

From the 46 species that encoded the Nan cluster, only 10 species also encoded the genes for the biosynthesis of sialic acid (*neuAB*) or nonulosonic acid (*nul*): *F. nucleatum, R. gnavus, C. botulinum, S. pealeana, S. agalactiae, V. fischeri*, *V. vulnificus, P. profundum*, and *Psychromonas*. There were only four species, *A. pleuropneumoniae, H. influenzae, H. somnus*, and *P. multocida *that encoded only NeuA, which is required for recognition of sialic acid by sialyltransferases and subsequent sialylation of the bacterial cell surface, suggesting that the donor-scavenging method of sialylation is limited. All the species that encoded *neuA *and *neuB *also encoded *neuC*, with one exception: *F. nucleatum*. As shown in other organisms, *Fusobacterium *might scavenge a precursor of sialic acid from its environment instead of synthesizing it de novo [49]. It is well known that some *E. coli *strains can synthesize sialic acid and sialylate their surface [3]. However the strains under study here do not encode *neuA *and *neuB*.

The distribution of the genes for the synthesis of sialic acid/nonulosonic acid in the Bacterial kingdom is very different from that of the Nan genes for catabolism. The sialic acid/nonulosonic acid synthesis genes are considerably more widespread both ecologically and taxonomically. For example, a high number of marine bacteria encode the Neu cluster such as species from the genera *Synechococcus*, *Salinibacter*, *Shewanella*, *Sphingopyxis*, *Chromobacterium*, *Hahella*, *Idiomarina*, *Prochlorococcus*, *Reinekea*, *Tenacibaculum*, *Rhodopseudomonas*, *Thiomicrospira *and most members of the family Vibrionaceae, *V. cholerae *is the notable exception.

### Colinearity of the Nan cluster

**Figure 2:**
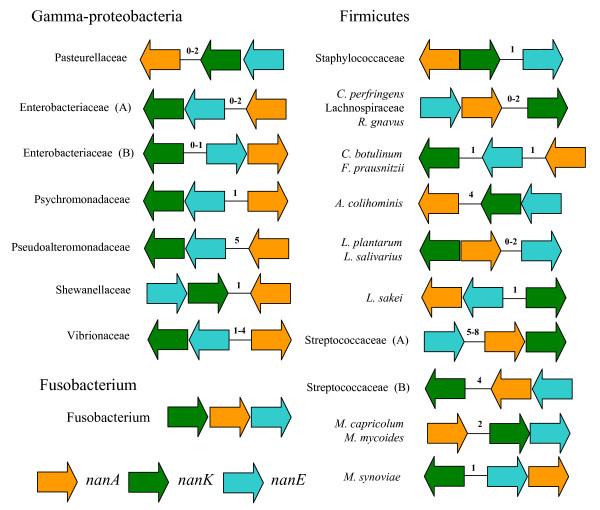
**Structure of the Nan clusters among bacterial groups**. Unless otherwise indicated all the members from a family shown share a common canonical structure of the Nan cluster, with differences only in the number of ORFs between the genes (indicated by a number within each cluster). *nanA*, orange arrows; *nanK*, green arrows; *nanE*, blue arrows. Enterobacteriaceae (A): *E. coli, S. enterica, S. typhimurium, S. boydii, S. dysenteriae, S. flexneri, S. sonnei*; Enterobacteriaceae (B): All species from genus *Yersinia*; Streptococcaceae (A) *S. agalactiae, S. gordonii, S. pneumoniae *(cluster A), (B) *S. pyogenes, S. sanguinis*, *S. pneumoniae *(cluster B).

**Figure 3:**
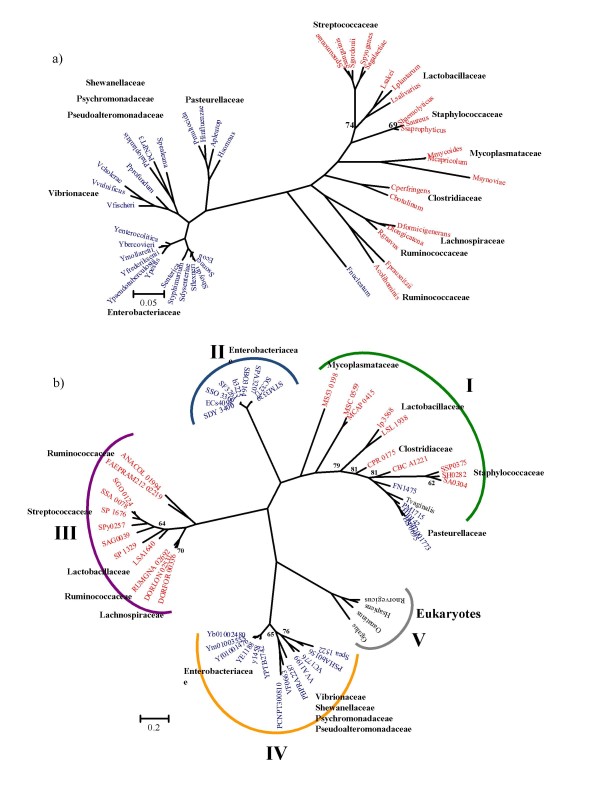
**Phylogenetic trees of a) 16S rRNA of bacteria containing the Nan cluster b) NanA**. The trees were obtained using Bayesian analysis as implemented in MrBayes. 1,000,000 generations were used to build the consensus trees. Only confidence values below 85 are shown. Blue Operational Taxonomic Units (OTUs) indicate Gram-negative Bacteria; red OTUs, Gram-positive Bacteria; black OTUs, Eukaryotes. For NanA tree the five main lineages are highlighted with color brackets embracing them. Lineage I, green bracket; lineage II, light blue bracket; lineage III, purple bracket; lineage IV, yellow bracket; lineage V, grey bracket.

The three main groups that contain the Nan cluster, Gamma-Proteobacteria, *Fusobacterium*, and Firmicutes, encode the *nanA*, *nanE*, and *nanK *genes in a different gene order and on different strands (Fig. 2). In fact, the gene order of the Nan cluster varies among families, and, to a lesser extent, within families (Fig. 2). Within the Gamma-Proteobacteria, there are seven variants of the Nan cluster, each family having its own gene order with the exception of the Enterobacteriaceae. Within this group,*Yersinia *species have a different gene order to *E. coli, Shigella*, and *S. enterica *(Fig. 2). Interestingly, in all species examined among the Gamma-Proteobacteria, except for *Yersinia*, the *nanK *and *nanE *genes are clustered together and encoded on the same strand, whereas the *nanA *gene is always separate from *nanE *and *nanK *by at least one gene (Fig. 2). Among the 20 Firmicutes examined, there are 10 different gene order combinations, but unlike Gamma-Proteobacteria, in only two cases did *nanE *and *nanK *cluster together. There is no clear canonical gene order within the Firmicutes, since the three genes cluster in almost all possible orders. This higher degree of variation of gene order in Firmicutes compared to the Gamma-Proteobacteria may reflect a long association within this diverse group (Fig. 3a).

As is well known, operons are pervasive in Prokaryotes [50]. However, the mechanisms underlying their evolution are not fully understood. Some authors argue that the fact that some genes are located within a single co-transcribed region selects for a more efficient regulation [51]. Also, a group of genes encoding co-dependent functions when forming an operon increases the likelihood of a fully functional horizontal gene transfer event, a major evolutionary force within bacterial evolution [51,52]. The Nan cluster would fall within what is considered a "destructed" operon, due to its loosely organized configuration, which might be due to rearrangements within its host genome or during the possible HGT events that led to its particular distribution, a scenario that is more widespread than previously thought [53,54]. Also it might indicate the relatively new acquisition of the genes by the bacterial kingdom, since fragmentation of well-adapted ancient operons will at least require the evolution of regulatory elements, which might not confer a selective advantage to the organism [55]. The latter hypothesis could explain why the Nan cluster is so limited in its distribution.

### Signatures of horizontal gene transfer

Typically, there are two main methods to detect putative horizontal gene transfer events: Phylogenetic methods and surrogate methods based on nucleotide composition. In addition, the presence of transposases and/or integrases within a region may suggest a mode of transfer. Therefore, we located all transposases and integrases within or near the Nan cluster (see Additional file 9). From the 46 species that encode the Nan cluster 12 species contained transposases close to the region, with a noticeable abundance in *C. perfringens *SM101, *L. salivarius *UCC118, *S. pneumoniae *TIGR B, and *Y. pestis *KIM (see Additional file 9). In *V. cholerae *N16961, the Nan region, is present on a pathogenicity island named Vibrio Pathogenicity Island-2 (VPI-2) and encodes an integrase [56]. Next, we compared the average GC content of the whole genome (GC_g_) against the GC content of the Nan genes (GC_nan_) shown as the difference between GC_g _and GC_nan _(see Additional file 7). None of the *nanA *gene sequences had a significant aberrant GC content (deviating from the GC_g _by +/- 5). However, *nanE *and *nanK *from *Yersinia spp*, *Shigella spp*, *E. coli, S. enterica, Pasteurella *and *H. somnus *had significant aberrant GC content suggesting its evolutionary history differs from *nanA*. In *S. pneumoniae *TIGR *nanE *and *nanK *from both clusters had an aberrant GC content; suggesting an independent history for the two clusters in this strain (see Additional file 7).

### Phylogenetic analysis of NanA

**Figure 4:**
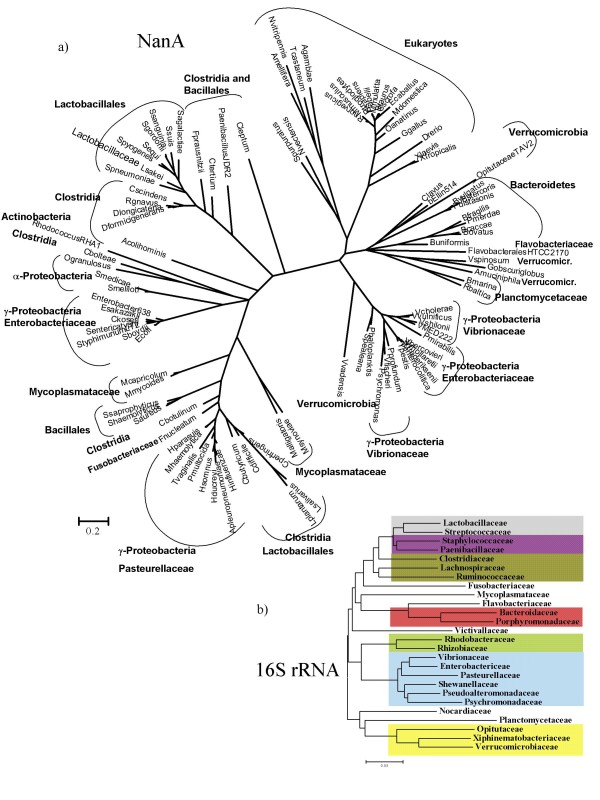
**Phylogenetic trees of a) All species encoding NanA b) 16S rRNA of all families encoding NanA**. The trees were obtained using Bayesian analysis as implemented in MrBayes. 1,000,000 generations were used to build the consensus trees as indicated in methods section. Main inclusive taxonomic groups are indicated. b) Grey shading, Lactobacillales; purple shading, Bacillales; dark green shading, Clostridia; red shading, Bacteroidetes; green shading, Alpha-proteobacteria; blue shading, Gamma-proteobacteria; yellow shading, Verrucomicrobia.

**Figure 5:**
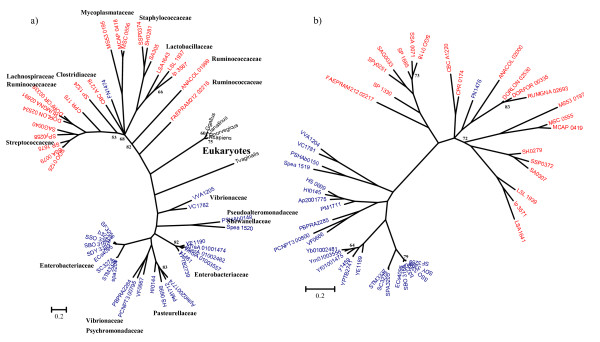
**Phylogenetic trees of a) NanK and b) NanE**. The trees were obtained using Bayesian analysis as implemented in MrBayes. 1,000,000 generations were used to build the consensus trees. Only confidence values below 85 are shown. Blue Operational Taxonomic Units (OTUs) indicate Gram-negative Bacteria; red OTUs, Gram-positive Bacteria; black OTUs, Eukaryotes. Blue branches indicate Gamma-proteobacteria; red branches, Firmicutes; Black branches; Eukaryotes.

The limited distribution of the Nan cluster, its variable gene order, and the diversity of transporters within the cluster, indicates mosaic evolution of the region (Fig. 2). In order to examine further the evolutionary history of the Nan region, we performed a phylogenetic analysis of NanA, NanE, and NanK and compared the branching patterns of the three proteins with the topology for the species tree based on 16S rRNA sequences (Figs. 3, 4 and 5).

The *nanA *gene encodes the enzyme *N*-acetylneuraminic lyase (NanA). As outgroups we included in our phylogenetic analysis NanA from four eukaryotes: *Gallus gallus*, *Ornithorhynchus anatinus*, *Rattus norvegicus*, and *Homo sapiens*. Overall, within the NanA phylogenetic tree, Gram-negative and Gram-positive representatives did not form two distinct lineages as in the 16S rRNA tree. Indeed, the NanA tree can be subdivided in five main lineages (named I, II, III, IV and V) (Fig. 3b), with Lineage I as the most divergent clustering, encompassing Gram-negative genera, *Actinobacillus, Haemophilus *and *Pasteurella*, Fusobacteria, Gram-positive genera, *Clostridium, Lactobacillus, Staphylococcus*, and *Mycoplasma *(Fig. 3b). Also within this lineage is NanA from *Trichomonas vaginalis*, a protozoan parasite that adheres to the urogenital tract, which branches with members of the family Pasteurellaceae and *Fusobacterium nucleatum*. The placement of *T. vaginalis *with members of the Pasteurellaceae and not the eukaryotes indicates interdomain transfer of NanA from an ancestor of Pasteurellaceae to a progenitor of *T. vaginalis*, which was noted previously by others [30,31]. *T. vaginalis *also encodes a homologue of NanK but does not encode NanE. Within lineage I are three members of the genus *Staphylococcus*, which cluster together, and within this branch is NanA from *C. botulinum *and *C. perfringens*. The NanA from *Lactobacillus salivarius *and *L. plantarum *branch together within lineage I whereas NanA from *L. sakei *is located within lineage III. All members of the genus *Mycoplasma *are found within lineage I (Fig. 3b). Members of the *Mycoplasma *are obligate pathogens found in a wide range of hosts, the primary habitats of human and animal mycoplasmas are mucous membranes of the respiratory and urogenital tracts, eyes, mammary glands and the joints [57]. The nine highly related NanA protein sequences from *Shigella spp*., *E. coli*, and *S. enterica*, gastrointestinal pathogens of humans form the separate lineage II (Fig. 3b).

The six Firmicutes not present within lineage I, cluster together within lineage III, *L. sakei*, branching firmly with *Streptococcus*, which suggests a common origin for NanA in this group. The five additional commensals of the human gut that contain NanA, *D. formicigenerans, D. longicatena*, *R. gnavus*, *A. colihominis *and *F. prausnitzii*, are also present within this lineage (Fig. 3b).

The NanA protein from members of the families Vibrionaceae, Shewanellaceae, Psychromonadaceae, and Pseudoaltermonadaceae are all located within the divergent lineage IV. Lineage IV branches away from both lineage I and II, which contain the other Gamma-Proteobacteria species examined. Thus, it appears that the origin of NanA in lineage IV is unique. The six *Yersinia *NanA sequences examined are also present within lineage IV and are unrelated to NanA from other species of Enterobacteriaceae suggesting that there is not a single origin for *nanA *among enterobacteria (Fig. 3a). Even more surprising is the close proximity of lineage IV to lineage V representing NanA from four eukaryotic species used as outgroups. A close relationship between NanA from *Vibrio/Yersinia *was previously indicated by Andersson and colleagues in their analysis of NanA from 10 bacterial species [30].

In order to investigate further the relationship between lineages IV and V, we reconstructed a phylogenetic tree including all sequences annotated as NanA in the genome database (Fig. 4 and Additional files 10 and 11). In total, we studied 83 putative NanA protein sequences from bacteria and an additional 18 NanA protein sequences from eukaryotes. From this analysis, six additional *Clostridium *species contain NanA (Fig. 4). Two human pathogens, *C. difficile *and *C. butyricum*, cluster with *C. botulinum, C. perfringens *and pathogenic species of Staphylococcaceae, Pasteurellaceae and Mycoplasmataceae (Fig. 4). Four *Clostridium *commensals cluster within three divergent branches, *C. leptum *forms a single divergent branch, *C. tertium *and *C. scindens *cluster with commensals from the family *Ruminococcaceae*, whereas *C. bolteae *clusters with the γ-Proteobacteria *Oceanicola granulosus *(Fig. 4). The distribution of NanA from the different *Clostridium *species on divergent branches of the tree indicates that in this species *nanA *was acquired multiple times and from different sources. Six additional γ-Proteobacteria species contained NanA, the majority of which are human commensals and pathogens (Fig. 4). A note of interest is the placement of NanA from the human commensal *Proteus mirabilis*, a member of the *Enterobacteriaceae*, firmly within the *Vibrionaceae *suggesting a recent acquisition from a *Vibrio *(Fig. 4).

NanA was present in two genera from α-Proteobacteria, and several members of the Bacteroidetes, Verrucomicrobia, and Planctomycetes. The NanA from the Gram-negative Bacteroidetes represents a large group of gut commensal (*Bacteroides caccae, B. fragilis, B. ovatus, B. stercoris, B. uniformis, B. vulgatus, Parabacteroides distasonis, P. merdae*, *Flavobacteriales bacterium*) that formed a distinct clade with five genera of the Verrucomicrobia and three genera of Planctomycetes. This clade was most closely related to the eukaryotes (Fig. 4). Planctomycetes are peptidoglycan-less bacteria with a shared compartmentalized cell structure and divide by a budding process [58]. Verrucomicrobia is a divergent phylum within the domain Bacteria, which also contains a compartmentalized cell structure similar to Planctomycetes [59]. It has been suggested that these groups, both inhabitants of the aquatic environment, may form some of the most ancient bacterial lineages (Fig. 4) [58,59]. Within the 16S rRNA tree the Bacteroidetes and Verrucomicrobia lineages are not related (Fig. 4). The NanA from the *Vibrio/Yersinia *lineage branches close to the Verrucomicrobia/Planctomycetes lineage and the eukaryote lineage (Fig. 4). A single species from the Verrucomicrobia also branches within the *Vibrio/Yersinia *clade. The data suggests horizontal transfer between eukaryotes and bacteria; however, the possible direction of transfer cannot be determined as the prokaryotes and eukaryotes form separate distinct clades (Fig. 4). We speculate that human pathogens such as *V. cholerae, V. vulnificus, Y. pestis *and *Y. enterocolitica *may have acquired *nanA *from a commensal species in the human gut. An alternative evolutionary scenario for this branching pattern may be convergent evolution of NanA, for instance, in order to recognize the same variant of sialic acid, which in the case of the bacterial pathogens within lineage IV would allow them to utilize the sialic acid found in the mucus of their host.

### Phylogenetic analysis of NanK and NanE

Unlike the NanA phylogeny where Gram-negative and Gram-positive species clustered together, the phylogenetic trees of both NanK and NanE did not demonstrate clear cases of horizontal transfer between the two groups (Fig. 5a and 5b). The NanE and NanK protein sequences from the Gamma-Proteobacteria branch separately from NanE and NanK from the Firmicutes. In both trees, *F. nucleatum *clusters firmly within the Firmicutes lineage (Fig. 5a and 5b). For the NanK tree we used the same outgroups as for the NanA tree, however, NanE is only present in bacteria. It is interesting to note that NanK from *T. vaginalis *does not cluster with the other eukaryotic representatives, which form a tight and closely related group, unrelated to any member of the bacterial kingdom.

Although in the NanE and NanK trees, Gamma-Proteobacteria and Firmicutes are grouped on separate lineages, similar to the 16S rRNA tree, within each lineage significant differences are found (Fig. 5). Strikingly, *P. profundum*, *V. fischeri*, and *Psychromonas *branch with members of the family Pasteurellaceae in both the NanK and NanE trees (Fig. 5a and 5b). *V. vulnificus *and *V. cholerae *are both closely related to each other in both trees but form a divergent lineage from the other members of the Vibrionaceae and Gamma-Proteobacteria strengthening the possibility of an independent evolutionary origin.

Within the Firmicutes lineages for the NanK protein there is significantly more diversity in the branching patterns than in the NanE tree (Fig. 5b). For both the NanE and NanK trees, the Staphylococci group branch with members of the genus *Lactobacillus*. The three members of *Mycoplasma *in the NanK tree form a divergent branch from other Firmicutes, similar to *F. nucleatum *and *C. botulinum*. NanK from *Streptococcus *species branch with *D. formicigenerans, D. longicatena*, and *R. gnavus*, similar to the NanA tree. The NanE and NanK proteins from the two species of the genus *Clostridium *are located on different branches in both trees (Fig. 5a and 5b).

Even though the general topologies of both the NanK and NanE trees resemble more closely the topology of the16S rRNA than that of NanA, there were many incongruencies found within the main lineages of the trees (Fig. 3, 4 and 5). These differences might be due to stochastic reasons, such as different rates of mutation between the two genes, or horizontal transfer events post speciation, which is suggested by the differences in the gene order of the clusters (Fig. 2). Within the majority of the Firmicutes *nanK *and *nanE *genes were not coupled together, whereas in Gamma-Proteobacteria the *nanK *and *nanE *genes were always coupled (one exception was noted within *Yersinia*). The coupling of the genes is likely reflected in the higher congruency between the topology for the Gamma-Proteobacteria for both NanE and NanK. The evolutionary scenario emerging from the analysis of the phylogenetic trees is a mosaic evolution of the Nan cluster, due to, among other possible reasons, horizontal transfer and reshuffling, as suggested by the variability in gene order.

### Utilization of sialic acid as a sole carbon source

**Figure 6:**
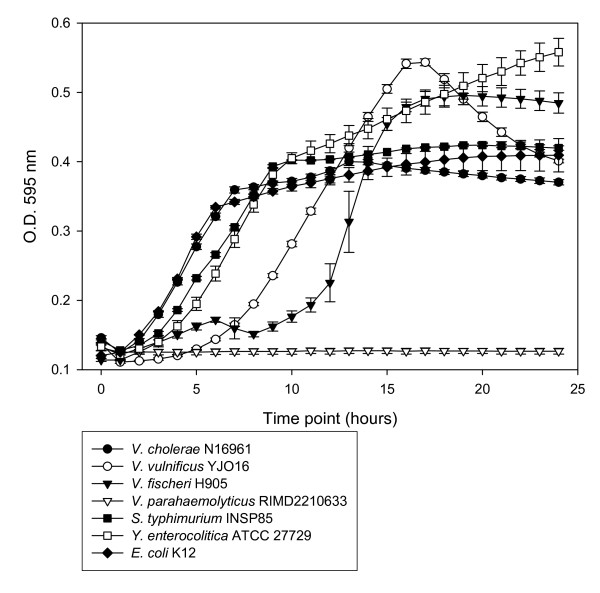
**Growth of species that encode the Nan cluster on minimal media supplemented with sialic acid**. Closed circles represent *V. cholerae *N16961; open circles represent *V. vulnificus *YJO16; closed triangles represent *V. fischeri *H905; open triangles represent *V. parahaemolyticus *RIMD2210633; closed squares represent *S. enterica *serovar Typhimurium INSP85; open squares represent *Y. enterocolitica *ATCC 27729; closed diamonds represent *E. coli *K12.

In order to verify whether the species predicted to encode the Nan cluster have the capability to survive using sialic acid as a sole carbon source, we performed in vitro assays studying the growth of some pathogenic and commensal bacteria on minimal media supplemented with sialic acid (M9+sialic acid) (Fig. 6). Previous studies have shown that some bacteria, such as *C. perfringens, B. fragilis, E. coli *K12, *P. multocida*, and *H. influenzae *can utilize sialic acid as a carbon source [15,22,23,27,29,60]. We examined growth of *V. cholerae *N16961, *V. vulnificus *YJO16, *V. fischeri *H905, *S. enterica *serovar Typhimurium INSP85, *Y. enterocolitica *ATCC 27729, *E. coli *K12 (positive control) and *V. parahaemolyticus *RIMD2210633 (negative control) on M9+sialic acid as a sole carbon source (Fig. 6). As expected all isolates grew with the exception of *V. parahaemolyticus *RIMD2210633, which does not contain the Nan region or NanA (Fig. 6). We also studied the growth of *Y. pestis *KIM D27 for 48 hours on M9 minimal media supplemented with amino acids, N-minimal media, and MOPS minimal media. Slight growth occurred only in MOPS minimal media when supplemented with sialic acid or D-glucose, with both substrates showing similar growth patterns (data not shown). It is worth noting that *Y. pestis *has a very slow growth rate and is an auxotroph for many nutrients. Overall our findings indicate that three major groups of pathogenic bacteria, *Salmonella, Vibrio *and *Yersinia*, can utilize sialic acid as a sole carbon and energy source, a nutrient widespread in the mucous surfaces colonized by these organisms, increasing their fitness in their host's environment.

## Conclusion

Sialobiology is a an emerging field in cellular microbiology, which is beginning to uncover the significant role of sialic acid metabolism in bacterial interactions with the human host both as commensals and pathogens. In this work, we studied the distribution, gene order, and molecular evolution of the cluster involved in sialic acid degradation (*nanA*, *nanE*, and *nanK*) among bacteria. We show for the first time that the Nan cluster is limited to pathogenic and commensal bacteria, encompassing a limited number of Gamma-Proteobacteria and Firmicutes. We demonstrated that NanA, the first enzyme in the catabolic pathway, has a distinct evolutionary history from NanE and NanK, with multiple instances of horizontal gene transfer found. The Nan cluster shows mosaic evolution with incongruencies in its phylogeny and diversity in its structure. For the first time, we confirm the predicted ability to utilize sialic acid as a carbon source in several bacterial pathogens encompassing three major groups *Salmonella, Vibrio *and *Yersinia*, which can provide them with a selective advantage in heavily sialylated environments such as the human gut.

## Authors' contributions

SAM and EFB designed research, analyzed data, and drafted the manuscript. SAM performed the research. Both authors read and approved the final manuscript.

## Supplementary Material

Additional file 1 **NanA NJ tree**. The figure shows a phylogenetic tree of NanA using Neighbor Joining as a tree building method. Click here for file

Additional file 2 **NanK NJ tree**. The figure shows a phylogenetic tree of NanK using Neighbor Joining as a tree building method. Click here for file

Additional file 3 **NanE NJ tree**. The figure shows a phylogenetic tree of NanE using Neighbor Joining as a tree building method. Click here for file

Additional file 4 **NanA ML tree**. The figure shows a phylogenetic tree of NanA using Maximum Likelihood as a tree building method. Click here for file

Additional file 5 **NanK ML tree**. The figure shows a phylogenetic tree of NanK using Maximum Likelihood as a tree building method. Click here for file

Additional file 6 **NanE ML tree**. The figure shows a phylogenetic tree of NanE using Maximum Likelihood as a tree building method. Click here for file

Additional file 7 **GC content differences between Nan genes and genome**. The table indicates the GC content differences between the Nan genes and the core genome of the bacterium where they are encoded. Click here for file

Additional file 8 **Distribution of Nan clusters among Bacteria**. The table indicates the bacterial species that encode a full version of the Nan cluster (*nanA*, *nanK*, and *nanE*) Click here for file

Additional file 9 **Presence of mobile elements and Neuraminidases**. The table indicates which species encode a neuraminidase, integrase and/or mobile elements in the proximities of the Nan cluster. Click here for file

Additional file 10 **NanA Bayesian All Bootstrap**. The figure shows a phylogenetic tree of NanA using Bayesian analysis as a tree building method with the bootstrap values indicated. Click here for file

Additional file 11 **Additional species included in Figure **4. The table indicates the extra additional species that were included in Fig. 4 that did not contain a full Nan cluster but encoded NanA. Click here for file

## References

[B1] Vimr ER, Kalivoda KA, Deszo EL, Steenbergen SM (2004). Diversity of microbial sialic acid metabolism. Microbiol Mol Biol Rev.

[B2] Angata T, Varki A (2002). Chemical diversity in the sialic acids and related alpha-keto acids: an evolutionary perspective. Chem Rev.

[B3] Severi E, Hood DW, Thomas GH (2007). Sialic acid utilization by bacterial pathogens. Microbiology.

[B4] Warren L (1994). Bound Carbohydrates in Nature.

[B5] Schauer R (2000). Achievements and challenges of sialic acid research. Glycoconj J.

[B6] Varki A (1993). Biological roles of oligosaccharides: all of the theories are correct. Glycobiology.

[B7] Schwarzkopf M, Knobeloch KP, Rohde E, Hinderlich S, Wiechens N, Lucka L, Horak I, Reutter W, Horstkorte R (2002). Sialylation is essential for early development in mice. Proc Natl Acad Sci USA.

[B8] Malykh YN, Krisch B, Gerardy-Schahn R, Lapina EB, Shaw L, Schauer R (1999). The presence of N-acetylneuraminic acid in Malpighian tubules of larvae of the cicada Philaenus spumarius. Glycoconj J.

[B9] Roth J, Kempf A, Reuter G, Schauer R, Gehring WJ (1992). Occurrence of sialic acids in Drosophila melanogaster. Science.

[B10] Saito M, Kitamura H, Sugiyama K (2001). Occurrence of gangliosides in the common squid and pacific octopus among protostomia. Biochim Biophys Acta.

[B11] Alviano CS, Travassos LR, Schauer R (1999). Sialic acids in fungi: a minireview. Glycoconj J.

[B12] Cross GA, Takle GB (1993). The surface trans-sialidase family of Trypanosoma cruzi. Annu Rev Microbiol.

[B13] Schauer R, Reuter G, Muhlpfordt H, Andrade AF, Pereira ME (1983). The occurrence of N-acetyl- and N-glycoloylneuraminic acid in Trypanosoma cruzi. Hoppe Seylers Z Physiol Chem.

[B14] Parsons NJ, Patel PV, Tan EL, Andrade JR, Nairn CA, Goldner M, Cole JA, Smith H (1988). Cytidine 5'-monophospho-N-acetyl neuraminic acid and a low molecular weight factor from human blood cells induce lipopolysaccharide alteration in gonococci when conferring resistance to killing by human serum. Microb Pathog.

[B15] Vimr E, Lichtensteiger C, Steenbergen S (2000). Sialic acid metabolism's dual function in Haemophilus influenzae. Mol Microbiol.

[B16] Vimr E, Steenbergen S, Cieslewicz M (1995). Biosynthesis of the polysialic acid capsule in Escherichia coli K1. J Ind Microbiol.

[B17] Vogel U, Claus H, Heinze G, Frosch M (1999). Role of lipopolysaccharide sialylation in serum resistance of serogroup B and C meningococcal disease isolates. Infect Immun.

[B18] Chang DE, Smalley DJ, Tucker DL, Leatham MP, Norris WE, Stevenson SJ, Anderson AB, Grissom JE, Laux DC, Cohen PS (2004). Carbon nutrition of Escherichia coli in the mouse intestine. Proc Natl Acad Sci USA.

[B19] Martinez J, Steenbergen S, Vimr E (1995). Derived structure of the putative sialic acid transporter from Escherichia coli predicts a novel sugar permease domain. J Bacteriol.

[B20] Schauer R, Buscher HP, Casals-Stenzel J (1974). Sialic acids: their analysis and enzymic modification in relation to the synthesis of submandibular-gland glycoproteins. Biochem Soc Symp.

[B21] Severi E, Randle G, Kivlin P, Whitfield K, Young R, Moxon R, Kelly D, Hood D, Thomas GH (2005). Sialic acid transport in Haemophilus influenzae is essential for lipopolysaccharide sialylation and serum resistance and is dependent on a novel tripartite ATP-independent periplasmic transporter. Mol Microbiol.

[B22] Steenbergen SM, Lichtensteiger CA, Caughlan R, Garfinkle J, Fuller TE, Vimr ER (2005). Sialic Acid metabolism and systemic pasteurellosis. Infect Immun.

[B23] Brigham C, Caughlan R, Gallegos R, Dallas MB, Godoy VG, Malamy MH (2009). Sialic Acid (N-acetyl Neuraminic Acid or NANA) Utilization by Bacteroides fragilis Requires A Novel N-acetyl Mannosamine (manNAc) Epimerase. J Bacteriol.

[B24] Comb DG, Roseman S (1960). The sialic acids. I. The structure and enzymatic synthesis of N-acetylneuraminic acid. J Biol Chem.

[B25] Plumbridge J, Vimr E (1999). Convergent pathways for utilization of the amino sugars N-acetylglucosamine, N-acetylmannosamine, and N-acetylneuraminic acid by Escherichia coli. J Bacteriol.

[B26] Ringenberg MA, Steenbergen SM, Vimr ER (2003). The first committed step in the biosynthesis of sialic acid by Escherichia coli K1 does not involve a phosphorylated N-acetylmannosamine intermediate. Mol Microbiol.

[B27] Vimr ER, Troy FA (1985). Regulation of sialic acid metabolism in Escherichia coli: role of N-acylneuraminate pyruvate-lyase. J Bacteriol.

[B28] Vimr ER, Troy FA (1985). Identification of an inducible catabolic system for sialic acids (nan) in Escherichia coli. J Bacteriol.

[B29] Nees S, Schauer R, Mayer F (1976). Purification and characterization of N-acetylneuraminate lyase from Clostridium perfringens. Hoppe Seylers Z Physiol Chem.

[B30] Andersson JO, Doolittle WF, Nesbo CL (2001). Genomics. Are there bugs in our genome?. Science.

[B31] de Koning AP, Brinkman FS, Jones SJ, Keeling PJ (2000). Lateral gene transfer and metabolic adaptation in the human parasite Trichomonas vaginalis. Mol Biol Evol.

[B32] Blattner FR, Plunkett G, Bloch CA, Perna NT, Burland V, Riley M, Collado-Vides J, Glasner JD, Rode CK, Mayhew GF (1997). The complete genome sequence of Escherichia coli K-12. Science.

[B33] Heidelberg JF, Eisen JA, Nelson WC, Clayton RA, Gwinn ML, Dodson RJ, Haft DH, Hickey EK, Peterson JD, Umayam L (2000). DNA sequence of both chromosomes of the cholera pathogen Vibrio cholerae. Nature.

[B34] Munson RS, Zhong H, Mungur R, Ray WC, Shea RJ, Mahairas GG, Mulks MH (2004). Haemophilus ducreyi strain ATCC 27722 contains a genetic element with homology to the vibrio RS1 element that can replicate as a plasmid and confer NAD independence on haemophilus influenzae. Infect Immun.

[B35] Larkin MA, Blackshields G, Brown NP, Chenna R, McGettigan PA, McWilliam H, Valentin F, Wallace IM, Wilm A, Lopez R (2007). Clustal W and Clustal X version 2.0. Bioinformatics.

[B36] Nicholas KBNH, Deerfield DW (1997). GeneDoc: Analysis and Visualization of Genetic Variation. EMBNEW.

[B37] Posada D, Crandall KA (1998). MODELTEST: testing the model of DNA substitution. Bioinformatics.

[B38] Lanave C, Preparata G, Saccone C, Serio G (1984). A new method for calculating evolutionary substitution rates. J Mol Evol.

[B39] Goldman N, Whelan S (2000). Statistical tests of gamma-distributed rate heterogeneity in models of sequence evolution in phylogenetics. Mol Biol Evol.

[B40] Felsenstein J (1989). Mathematics vs. Evolution: Mathematical Evolutionary Theory. Science.

[B41] Huelsenbeck JP, Ronquist F (2001). MRBAYES: Bayesian inference of phylogenetic trees. Bioinformatics.

[B42] Guindon S, Gascuel O (2003). A simple, fast, and accurate algorithm to estimate large phylogenies by maximum likelihood. Syst Biol.

[B43] Tamura K, Dudley J, Nei M, Kumar S (2007). MEGA4: Molecular Evolutionary Genetics Analysis (MEGA) software version 4.0. Mol Biol Evol.

[B44] Huson DH, Bryant D (2006). Application of phylogenetic networks in evolutionary studies. Mol Biol Evol.

[B45] Karlin S (2001). Detecting anomalous gene clusters and pathogenicity islands in diverse bacterial genomes. Trends Microbiol.

[B46] Brubaker RR (1970). Interconversion of Purine Mononucleotides in Pasteurella pestis. Infect Immun.

[B47] Kuwahara T, Yamashita A, Hirakawa H, Nakayama H, Toh H, Okada N, Kuhara S, Hattori M, Hayashi T, Ohnishi Y (2004). Genomic analysis of Bacteroides fragilis reveals extensive DNA inversions regulating cell surface adaptation. Proc Natl Acad Sci USA.

[B48] Post DM, Mungur R, Gibson BW, Munson RS (2005). Identification of a novel sialic acid transporter in Haemophilus ducreyi. Infect Immun.

[B49] Vimr E, Lichtensteiger C (2002). To sialylate, or not to sialylate: that is the question. Trends Microbiol.

[B50] Fani R, Brilli M, Lio P (2005). The origin and evolution of operons: the piecewise building of the proteobacterial histidine operon. J Mol Evol.

[B51] Lawrence JG, Roth JR (1996). Selfish operons: horizontal transfer may drive the evolution of gene clusters. Genetics.

[B52] Woese C (1998). The universal ancestor. Proc Natl Acad Sci USA.

[B53] Dandekar T, Snel B, Huynen M, Bork P (1998). Conservation of gene order: a fingerprint of proteins that physically interact. Trends Biochem Sci.

[B54] Itoh T, Takemoto K, Mori H, Gojobori T (1999). Evolutionary instability of operon structures disclosed by sequence comparisons of complete microbial genomes. Mol Biol Evol.

[B55] Omelchenko MV, Makarova KS, Wolf YI, Rogozin IB, Koonin EV (2003). Evolution of mosaic operons by horizontal gene transfer and gene displacement in situ. Genome Biol.

[B56] Jermyn WS, Boyd EF (2002). Characterization of a novel Vibrio pathogenicity island (VPI-2) encoding neuraminidase (nanH) among toxigenic Vibrio cholerae isolates. Microbiology.

[B57] Vasconcelos AT, Ferreira HB, Bizarro CV, Bonatto SL, Carvalho MO, Pinto PM, Almeida DF, Almeida LG, Almeida R, Alves-Filho L (2005). Swine and poultry pathogens: the complete genome sequences of two strains of Mycoplasma hyopneumoniae and a strain of Mycoplasma synoviae. J Bacteriol.

[B58] Lee KC, Webb RI, Fuerst JA (2009). The cell cycle of the planctomycete Gemmata obscuriglobus with respect to cell compartmentalization. BMC Cell Biol.

[B59] Lee KC, Webb RI, Janssen PH, Sangwan P, Romeo T, Staley JT, Fuerst JA (2009). Phylum Verrucomicrobia representatives share a compartmentalized cell plan with members of bacterial phylum Planctomycetes. BMC Microbiol.

[B60] Allen S, Zaleski A, Johnston JW, Gibson BW, Apicella MA (2005). Novel sialic acid transporter of Haemophilus influenzae. Infect Immun.

