# Supplementary figures and images for: Insights into the evolution of sialic acid catabolism among bacteria
Source: BMC Evol Biol. 2009 May 26;9:118. doi: 10.1186/1471-2148-9-118 (PMC2693436; doi:10.1186/1471-2148-9-118)

# NanA NJ tree

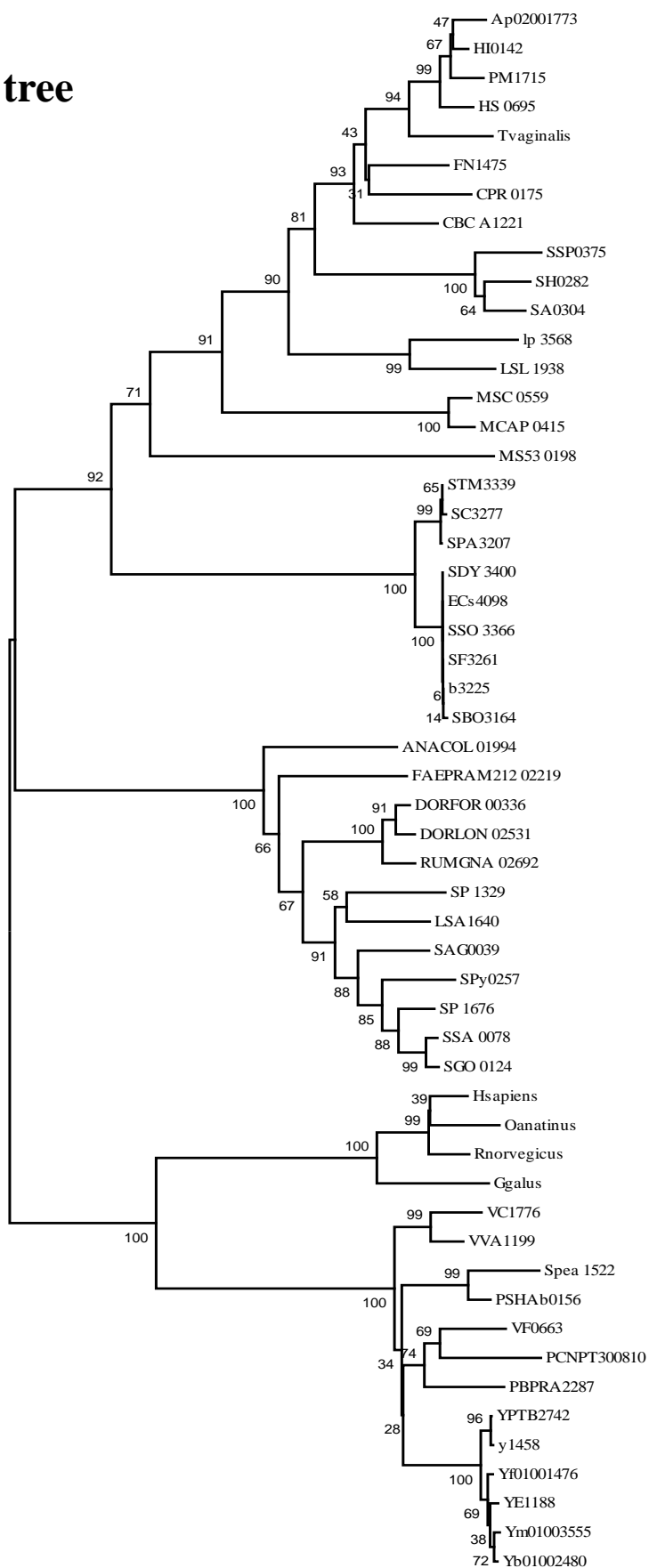

0.1

Supplement: Additional file 1 — NanA NJ tree. The figure shows a phylogenetic tree of NanA using Neighbor Joining as a tree building method. [file 1471-2148-9-118-S1.pdf]

Nank NJ tree

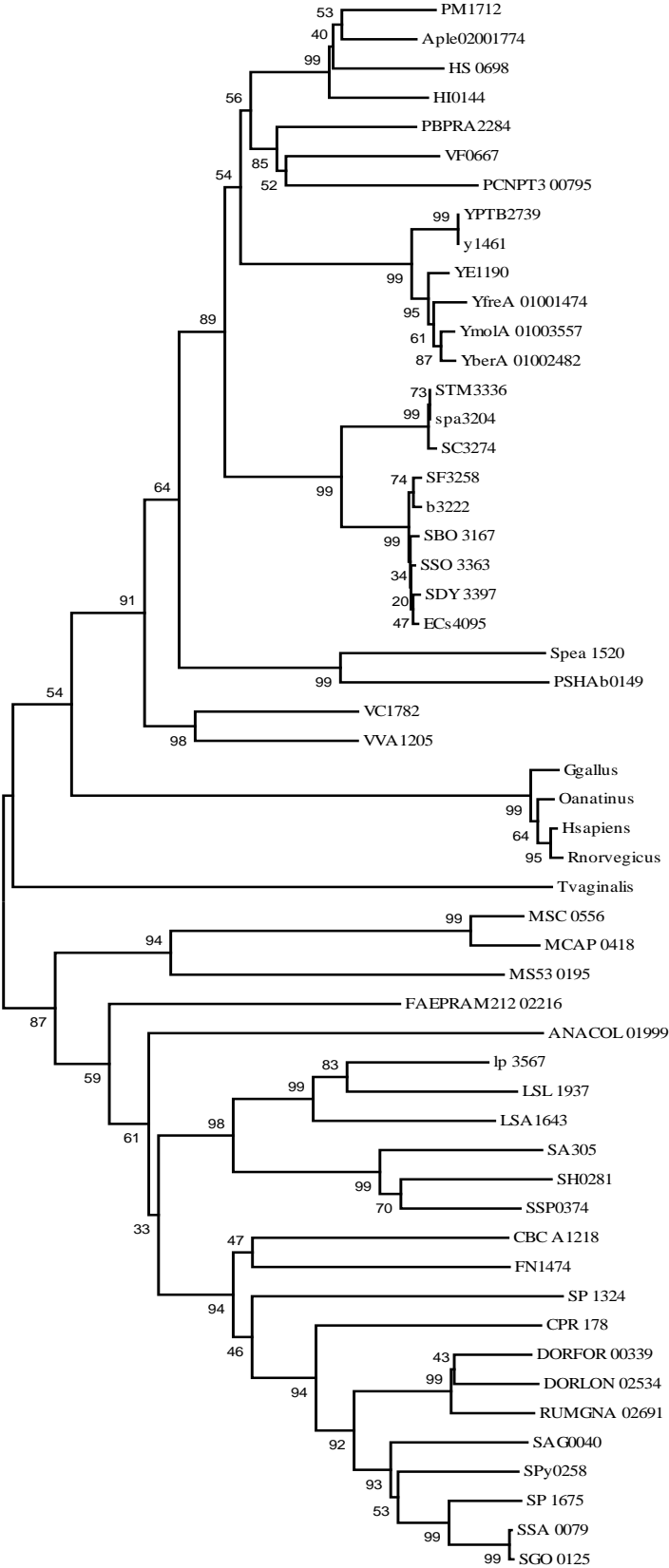

0.1

Supplement: Additional file 2 — NanK NJ tree. The figure shows a phylogenetic tree of NanK using Neighbor Joining as a tree building method. [file 1471-2148-9-118-S2.pdf]

NanE NJ tree

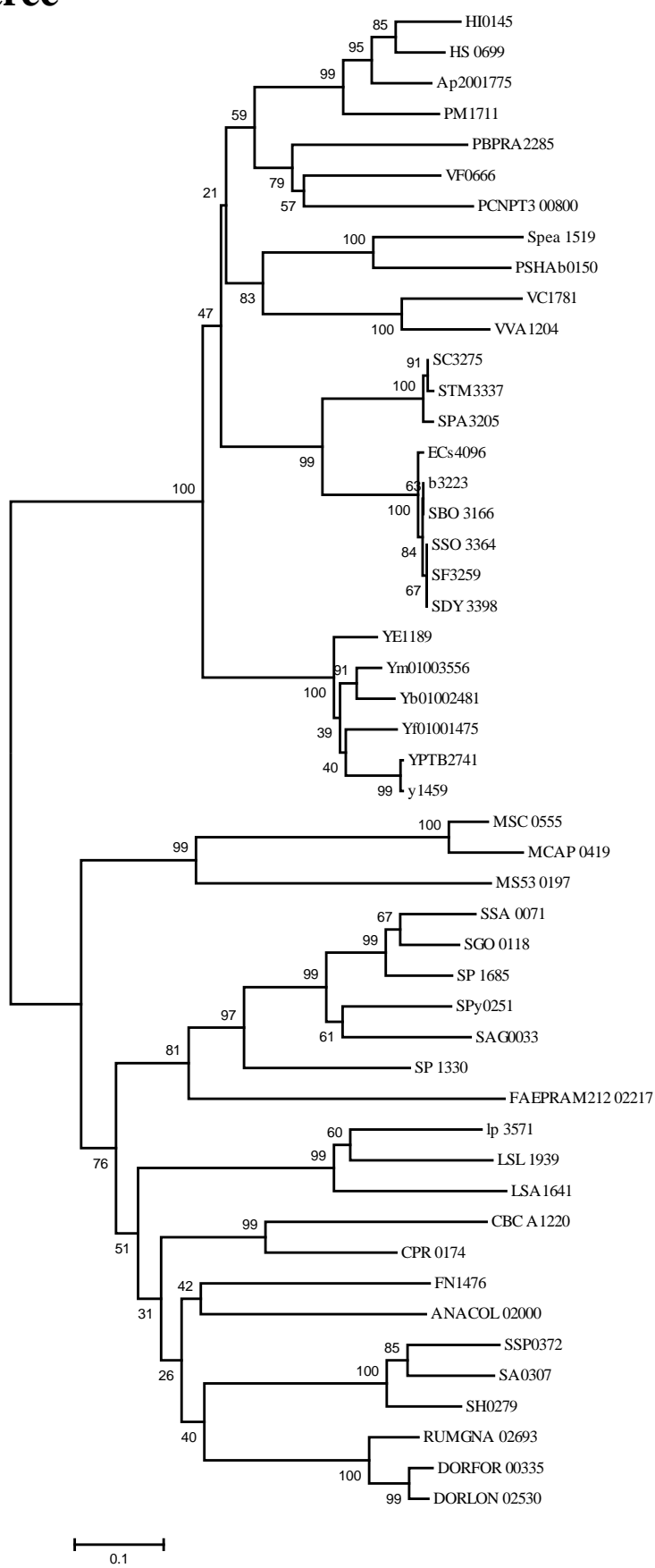

Supplement: Additional file 3 — NanE NJ tree. The figure shows a phylogenetic tree of NanE using Neighbor Joining as a tree building method. [file 1471-2148-9-118-S3.pdf]

# NanA ML tree

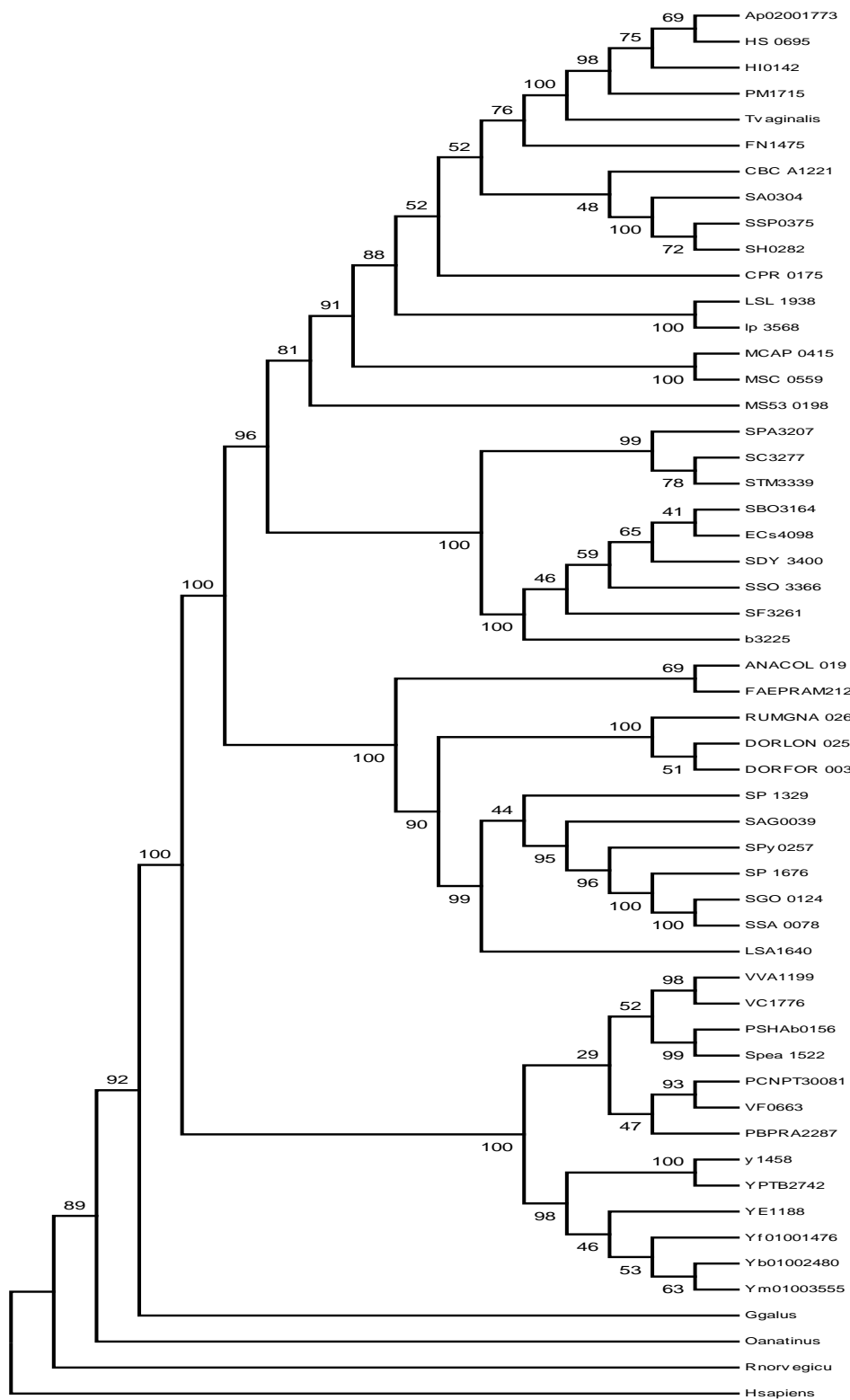

Supplement: Additional file 4 — NanA ML tree. The figure shows a phylogenetic tree of NanA using Maximum Likelihood as a tree building method. [file 1471-2148-9-118-S4.pdf]

# NanK ML tree

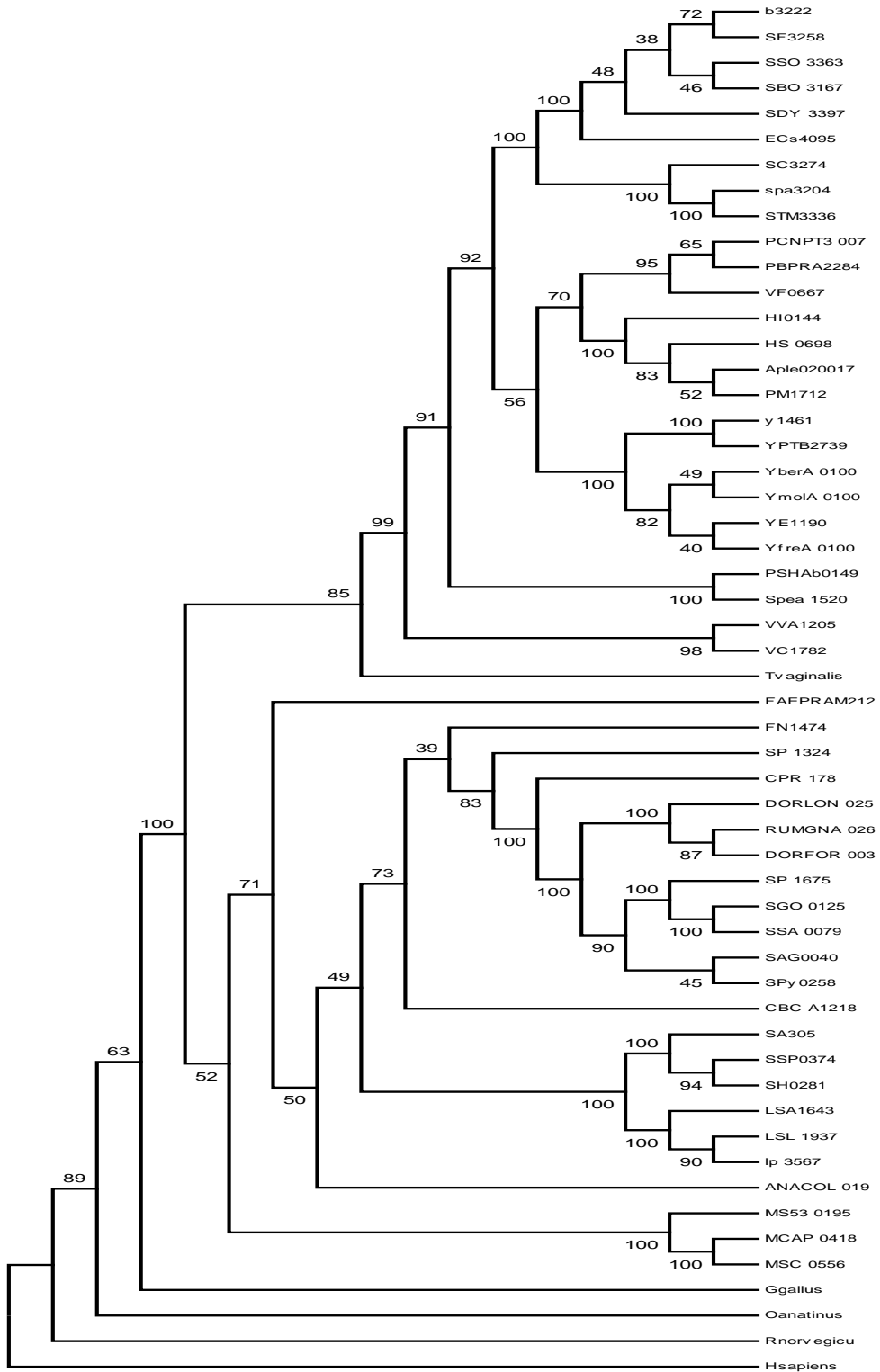

Supplement: Additional file 5 — NanK ML tree. The figure shows a phylogenetic tree of NanK using Maximum Likelihood as a tree building method. [file 1471-2148-9-118-S5.pdf]

# NanE ML tree

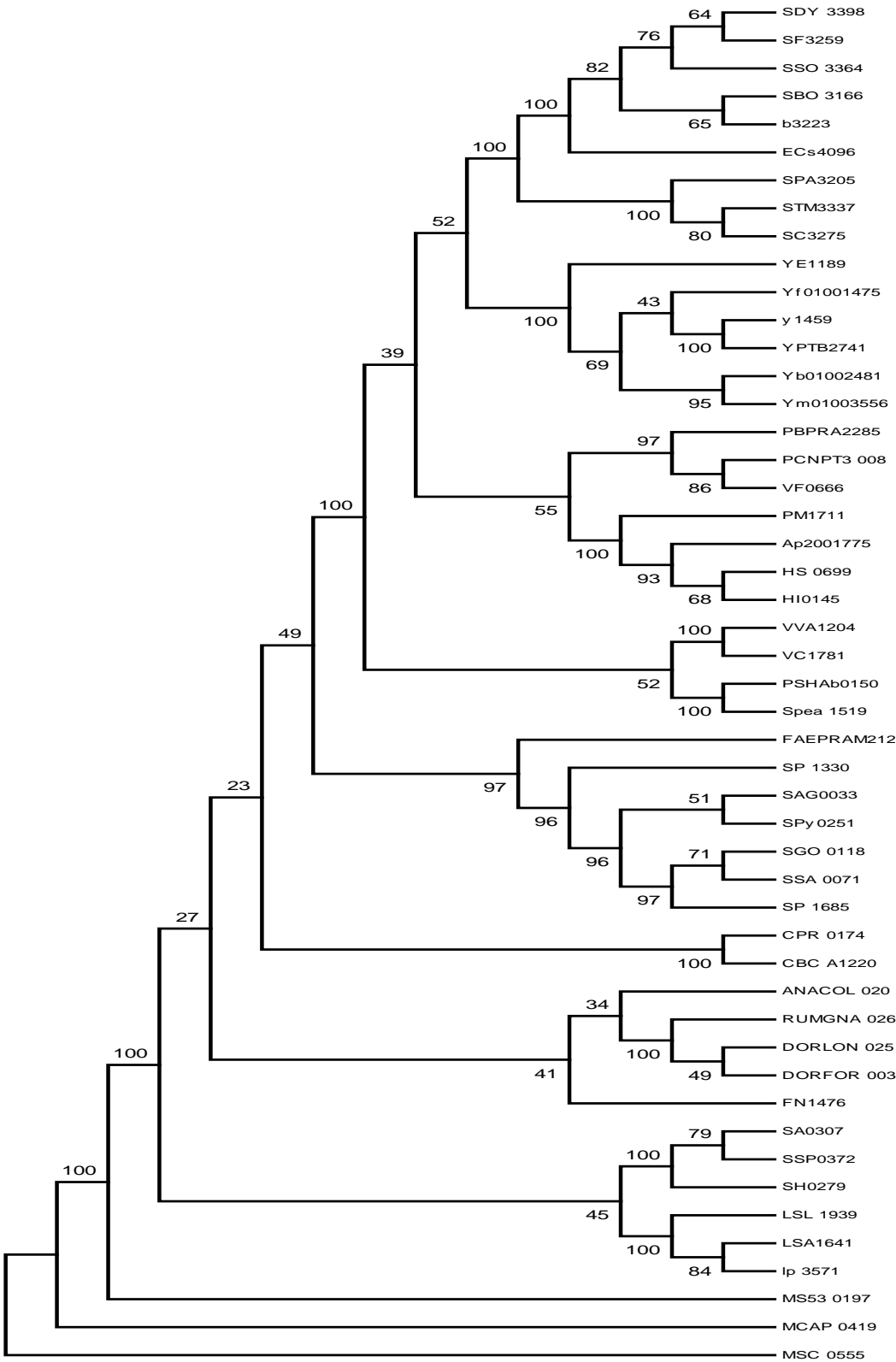

Supplement: Additional file 6 — NanE ML tree. The figure shows a phylogenetic tree of NanE using Maximum Likelihood as a tree building method. [file 1471-2148-9-118-S6.pdf]

# NanA Bayesian bootstrap

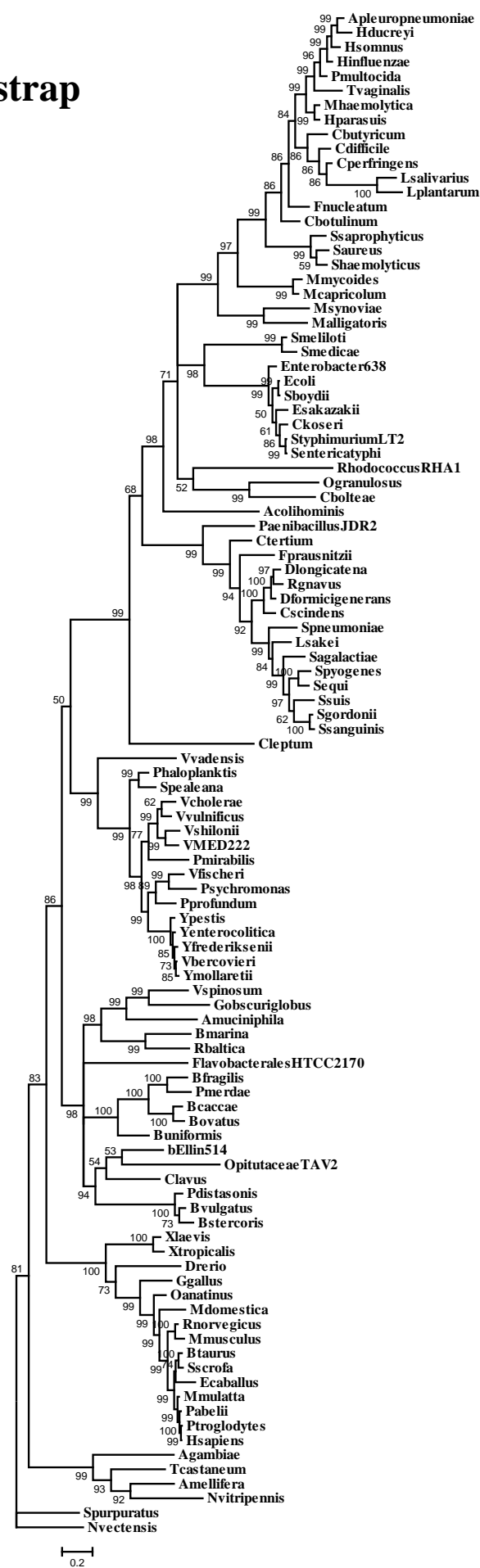

Supplement: Additional file 10 — NanA Bayesian All Bootstrap. The figure shows a phylogenetic tree of NanA using Bayesian analysis as a tree building method with the bootstrap values indicated. [file 1471-2148-9-118-S10.pdf]
